# Emerging Role of Radiologist in Multidisciplinary Team Meetings

**DOI:** 10.12669/pjms.39.4.7645

**Published:** 2023

**Authors:** Sadaf Arooj

A Multidisciplinary Team Meeting (MDT) is defined as a group of specialists intended to discuss management of cancer patients. MDT is the best practice for patient management.^1^ It is defined by the National Cancer Institute as a “treatment planning approach in which a number of doctors who are experts in different specialties review and discuss the medical condition and treatment options of a patient”.^2^ MDTs were started in 1995 and later became an essential part of Cancer Plan of National Health Service. Composition of an MDT can be variable according to hospital setting, however it usually consists of consultants from departments of Oncology, Surgery, Pathology and Radiology.^3^

Radiology is center of clinical diagnosis and patient care. It has become epitome of medical and surgical management. Be it an anomaly scan/obstetrical ultrasound or prompt reporting of MRI pelvis for rectal cancer staging, it plays pivotal role for early identification of the disease as well as further planning of patient management. There has been substantial increase in imaging technology in past two decades, followed by resultant increased cross sectional imaging including CT scan and MRI. Thus, radiologists are mandatory participants of MDTs.^4^

In 2005 Royal College of Radiologists (RCR) guidelines were presented for radiologists participating in MDTs. Later on, these were reformulated as 15 standards, divisible in five topics including time requirement, quality control, record keeping and facilities of job planning/appraisal.^5^ Meanwhile, radiologists working globally started complaining about increasing workload and found difficulties as far as the administration and time management were concerned. These had led to decrease in other academic activities within the Radiology Departments which were already catering not only clinical services to all departments but also teaching and training undergraduates, interns and post graduates.

In 2020 an audit was done and all radiologist at the United Kingdom were included. It was published recently by Elsevier on behalf of RCR. It was the study that achieved highest response rate for all over UK. The audit results summarized what problems radiologists were facing e.g. non availability of referral pro forma, pre MDT meetings as well as increased number of patients leading to decreased overall preparation time especially for patients bringing outside imaging investigations. In 56% cases no record of MDT discussion was available.^6^

EOSI (European Society of Oncologic Imaging) also conducted a survey to evaluate the role of radiologist and extent of their involvement in the MDTs. A total of 292 members participated. Two sixty radiologists were attending the MDTs in their hospitals. Out of these, only 173 would receive formal list of patients to be discussed with imaging investigations to review while 33 received only the list of patients and no imaging. Fifty were unable to prepare before time as they were not formally invited nor they received any images or lists. The importance of role of radiologist as core member of MDT can be estimated by the facts that in 40% cases discussed at MDTs, the report of prior radiologist was changed by the core member leading to altered clinical treatment offered. These patients would require a supplementary report. Survey also emphasized the benefits of MDTs including translational research, information regarding ongoing clinical research trials in hospitals, improvement in knowledge about cancer management, better interdepartmental collaboration and feedback. The deficiencies of ongoing MDTs were less clear clinical question, absence of referring physician, inadequacy of IT services provided, insufficient provision of documents/files of patients, odd timings and lack of time to attend these meetings.^7^

Nasir et al reviewed the role of radiologist and MDT workload. They evaluated that for a total of 223 meetings held over 15 months, preparation time by Senior Registrar was 47.93hours/month which is highly significant. Moreover, 1120 cases were discussed in these along with 2759 imaging studies showing the burden on radiologist.^8^

Contribution of MDTs to patient management is precise staging of cancer and early decision of neo/adjuvant chemotherapy. These need to be standardized globally. In our part of the world most of the tertiary care hospitals are conducting MDTs but no special way of electronic record keeping and other resources are available. Further improvement can be brought by providing these IT/PACS (picture archiving communication system) facilities and optimizing radiologist workload by increasing workforce in department for continuous and standardized service provision to patients along with research and training.
